# Human Kinase/Phosphatase-Wide RNAi Screening Identified Checkpoint Kinase 2 as a Cellular Factor Facilitating Japanese Encephalitis Virus Infection

**DOI:** 10.3389/fcimb.2018.00142

**Published:** 2018-05-17

**Authors:** Yi-Lin Chan, Ching-Len Liao, Yi-Ling Lin

**Affiliations:** ^1^Department of Life Science, Chinese Culture University, Taipei, Taiwan; ^2^Institute of Biomedical Sciences, Academia Sinica, Taipei, Taiwan; ^3^Department of Microbiology and Immunology, National Defense Medical Center, Taipei, Taiwan; ^4^National Institute of Infectious Diseases and Vaccinology, National Health Research Institutes, Miaoli, Taiwan; ^5^Genomics Research Center, Academia Sinica, Taipei, Taiwan

**Keywords:** Japanese encephalitis virus (JEV), kinase/phosphatase-wide RNAi screening, checkpoint kinase 2 (CHK2), ataxia-telangiectasia mutated kinase (ATM), cell cycle G1 arrest

## Abstract

Japanese encephalitis virus (JEV), a mosquito-borne flavivirus, causes acute encephalitis in humans with high mortality. Not much is known about the interactions between viral and cellular factors that regulate JEV infection. By using a kinase/phosphatase-wide RNAi screening approach, we identified a cell cycle-regulating molecule, checkpoint kinase 2 (CHK2), that plays a role in regulating JEV replication. JEV infection induced G1 arrest and activated CHK2. Inactivation of CHK2 and its upstream ataxia-telangiectasia mutated kinase in JEV-infected cells by using inhibitors reduced virus replication. Likewise, JEV replication was significantly decreased by knockdown of CHK2 expression with shRNA-producing lentiviral transduction. We identified CHK2 as a cellular factor participating in JEV replication, for a new strategy in addressing JEV infection.

## Introduction

Japanese encephalitis virus (JEV) is the most important cause of viral encephalitis in Southeast Asia, with 30,000–50,000 cases reported annually (Hegde and Gore, 2017). JEV is transmitted in a zoonotic cycle between mosquitoes and vertebrate-amplifying hosts, mainly swine and wading birds (Mansfield et al., 2017). As a member of the *Flavivirus* genus, the JEV virion is enveloped and has a positive-sense single-stranded RNA genome. The initial steps of JEV infection include virus attachment to cell-surface receptors and entry via receptor-mediated endocytosis. Translation of the viral genome produces a polyprotein that is processed to structural core (C), precursor of membrane (prM), and envelope (E) proteins and the nonstructural proteins NS1~NS5. Flaviviral genome replication occurs by the viral replicase complex via RNA-dependent RNA polymerization. The positive-sense genomic RNA is transcribed to a replication-intermediate negative-sense RNA, which is then used as a template to synthesize genomic RNA for subsequent translation and assembly of virion progeny (Tiroumourougane et al., 2002; Fields et al., 2007).

How a virus triggers DNA damage signaling is not fully understood, but previous reports have suggested that the cellular DNA repair machinery can recognize viral genetic materials, such as replicating nucleic acids and viral proteins, upon infection (Weitzman et al., 2004). Some viruses have been shown to interact with and/or affect components of the ATM DNA damage pathway (Lilley et al., 2007; Bagga and Bouchard, 2014). DNA viruses, such as human cytomegalovirus (CMV) activate the ATM checkpoint pathway during DNA replication and inhibit DNA damage responses by mislocalizing checkpoint proteins from the nucleus to cytoplasm (Gaspar and Shenk, 2006). Herpes simplex virus (HSV) induces an ATM-damage response that is essential for viral replication (Lilley et al., 2005; Shirata et al., 2005). Inhibition of CHK2 kinase activity by the CHK2 inhibitor II significantly reduces the CPE and genome replication of HSV-1 in corneal epithelium (Alekseev et al., 2015). Hepatitis C virus (HCV), an RNA virus belonging to *Flaviviridae*, induces G2/M phase arrest (Wu et al., 2008) and activates the dsDNA damage response pathway by causing DSBs and enhancing the mutation of cellular genes (Machida et al., 2004). Knockdown of ATM or CHK2 suppresses RNA replication of HCV (Ariumi et al., 2008), and HCV NS3-NS4A interacts with ATM, whereas HCV NS5B interacts with both ATM and CHK2 (Lai et al., 2008). JEV is able to manipulate the cell cycle, which reduces the proliferation of neural progenitor cells (Das and Basu, 2008) and allows for persistent infection (Kim et al., 2015). JEV is also able to manipulate the cell cycle, which reduces the proliferation of neural progenitor cells (Das and Basu, 2008) and allows for persistent infection (Kim et al., 2015).

Only a few host proteins have been found involved in JEV infection, with the roles in JEV replication remaining largely unknown. In this study, we identified CHK2, which participates in cellular regulation of JEV infection, by using a kinase/phosphatase-wide siRNA screening strategy. Both CHK2 activation and G1 phase arrest were noted in JEV infected cells and the activation of CHK2 appeared to be beneficial for JEV replication.

## Materials and methods

### Cell lines and reagents

U87 cells (ATCC HTB-14), a human glioblastoma cell line, were cultured in minimal essential medium (Gibco) containing 10% fetal bovine serum (FBS), 1% sodium pyruvate, and 2 mM L-glutamine. A549 cells, a human lung carcinoma cell line, were cultured as previously described (Chang et al., 2006). BE(2)C (ATCC CRL68), a human neuroblastoma cell line were cultured in10% FBS and 2 mM L-glutamine containing RPMI 1640 (Gibco) medium. HEK293T, a human embryonic kidney cells, were cultured in 10% FBS and 2 mM L-glutamine containing Dulbecco's Modified Eagle's Medium (Gibco). Experiments were done using cells with passage 3–5. The ATM inhibitor (KU-55933) was from Calbiochem and the CHK2 inhibitor II was from Merck.

### Viruses and viral infection

JEV strain RP-9 (Chen et al., 1996) was propagated in C6/36 cells. In Taiwan, JEV is classified as a BSL-2 pathogen according to the “Guidelines for research involving recombinant DNA molecules” issued by National Science Council, Taiwan (NSC., 2004). For viral infection, cells were adsorbed with viruses at the indicated multiplicity of infection (MOI) for 1 h at 37°C. Virus titers (PFU/ml) were determined by plaque-forming assays by using BHK-21 cells as described (Wu et al., 2002).

### Human kinase/phosphatase-wide RNAi screening

U87 cells were transduced with VSV-G pseudotyped lentiviruses expressing shRNAs targeting 1,260 human kinases and phosphatases (obtained from the National RNAi Core Facility, Taiwan). After puromycin selection, cells were infected with JEV and surviving cell colonies were cultured. Genomic DNAs from surviving cells were extracted by using the QIAamp DNA Mini and Blood Mini Kit (Qiagen). The DNA regions containing shRNAs were amplified by PCR with primers (5′-TAATTTCTTGGGTAGTTTGCAGTT-3′ and 5′-CCCCAATCCCCCCTTTTC-3′) and cloned into pcDNA3.1/V5-His-TOPO (Invitrogen). The genes targeted by shRNA were identified by sequencing and BLAST search.

### Lentivirus preparation

Lentiviral vectors (pLKO.1-puro) carrying the shRNAs targeting CHK2 (5′-GAACAGATAAATACCGAACAT-3′) and control LacZ (5′-TGTTCGCATTATCCGAACCAT-3′) were co-transfected into 293T cells with pMD.G and pCMVΔR8.91 (obtained from the National RNAi Core Facility, Taiwan) by using lipofectamine 2000 reagent. The culture supernatants were harvested and further concentrated by centrifugation at 20,000 × g for 3 h at 4°C.

### Cell survival

Cells were collected for survival determination by a trypan blue exclusion method. The virus-induced cytopathic effect (CPE) was analyzed by measuring the release of a cytoplasmic enzyme, lactate dehydrogenase (LDH), by using a commercial kit (Cytotoxicity Detection Kit; Roche).

### Immunoblotting

Cells were lysed and subjected to western blot analysis as described (Chan et al., 2008). Primary antibodies used in this study included anti-CHK2 (sc5278, Santa Cruz Biotechnology; H00011200-M01, Abnova), anti-CHK2 (Thr68) (#2661, Cell Signaling Technology), and anti-β actin (Chemicon). The mouse monoclonal antibody specific to JEV NS3 was described previously (Wu et al., 2002).

### RNA preparation and RT-PCR analysis

RNA was extracted by using an RNeasy kit (Qiagen) according to the manufacturer's instructions. cDNA was reverse transcribed from 2 μg RNA with a random hexamer and a ThermoScript RT Kit (Invitrogen). PCR involved specific primer sets for CHK2 (5′-ATGTCTCGGGAGTCGGATGTT-3′ and 5′-ACTTTATTTCTGCTTAGTGACAGTGCA-3′) and β-actin (5′-TCCTGTGGCATCCACGAAACT-3′ and 5′-GAAGCATTTGCGGTGGACGAT-3′), respectively.

### Cell cycle analysis by flow cytometry

The cell cycle status was analyzed by propidium iodide (PI) staining for nuclear DNA contents and by flow cytometry. At the indicated times after viral infection, cells were collected and fixed with 70% ethanol. Cells were stained with PI solution containing RNaseA at 4°C for 30 min, and then analyzed by flow cytometry (Attune NxT, Thermo Fisher).

### Statistical analysis

Data are presented as mean ± standard deviation (SD). Two categorical data were compared by independent Student *t-*test. The statistical tests were two-tailed and significance was set at *P* < 0.05, < 0.01, and < 0.005. For immunoblotting, the band density was quantified by use of ImageJ (US National Institutes of Health).

## Results

### Human kinase/phosphatase-wide RNAi screening identified CHK2 as a cellular factor involved in JEV infection

**Figure 1:**
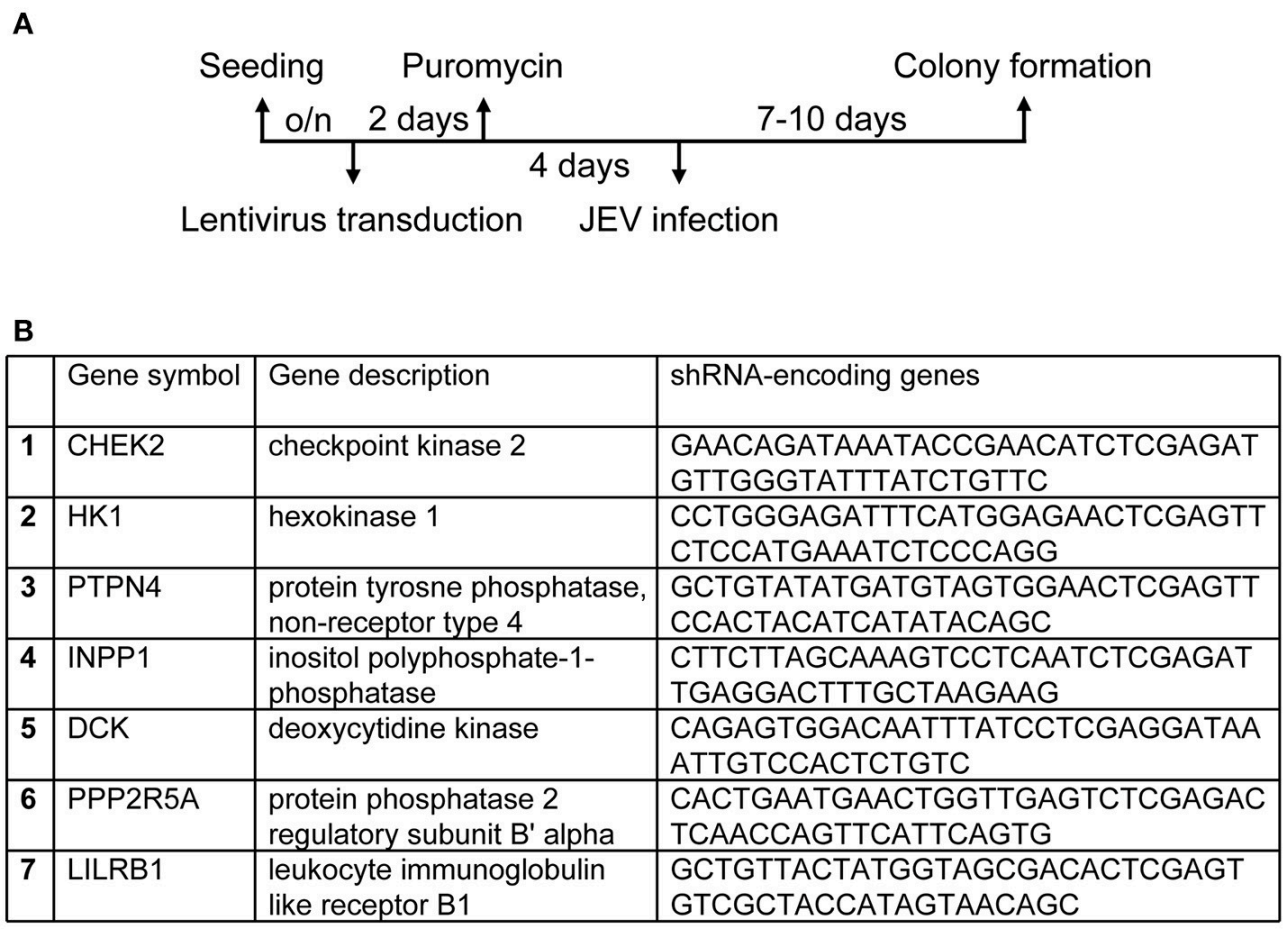
Establishing a human kinases/phophatases-wide RNAi screen system. **(A)** Overview of RNAi screening to genes involved in regulation of JEV infection. U87 cells transduced with lentiviruses expressing shRNAs targeting human kinases and phosphatase were selected with puromycin (10 μg/ml) for 4 days and infected JEV at an MOI of 10. **(B)** Cells survived from JEV infection were identified for candidate genes.

We used a human kinase/phosphatase-wide RNAi screening strategy to search for potential kinases and phosphatases involved in JEV infection. U87, a human glioma cell line, was transduced by each of the seven VSV-G pseudotyped lentivirus pool (Human kinase and phosphatase set) provided by the National RNAi Core Facility. Each kinases/phosphatases pooled tube contains ~180 kinase/phosphatase genes; each gene is targeted by 5 shRNAs that bind to distinct target sequences. The VSV-G pseudotyped lentivirus set that carries these shRNAs knocked down 1260 genes encoding kinase/phosphatases, which accounts for ~90% of all kinase/phosphatase in accordance of the NCBI database. After selection with puromycin for lentivirus-transduced cells, cells were infected JEV at an MOI of 10 (Figure 1A). Surviving cell colonies were cultured to extract genomic DNA. DNAs encoding shRNA were amplified by PCR and sequenced to determine their targets by BLAST alignment with the NCBI database to further confirm the identities of these genes as kinase/phosphatase encoding genes. Seven host candidate genes (Figure 1B), were identified in cells survived from JEV challenge.

**Figure 2:**
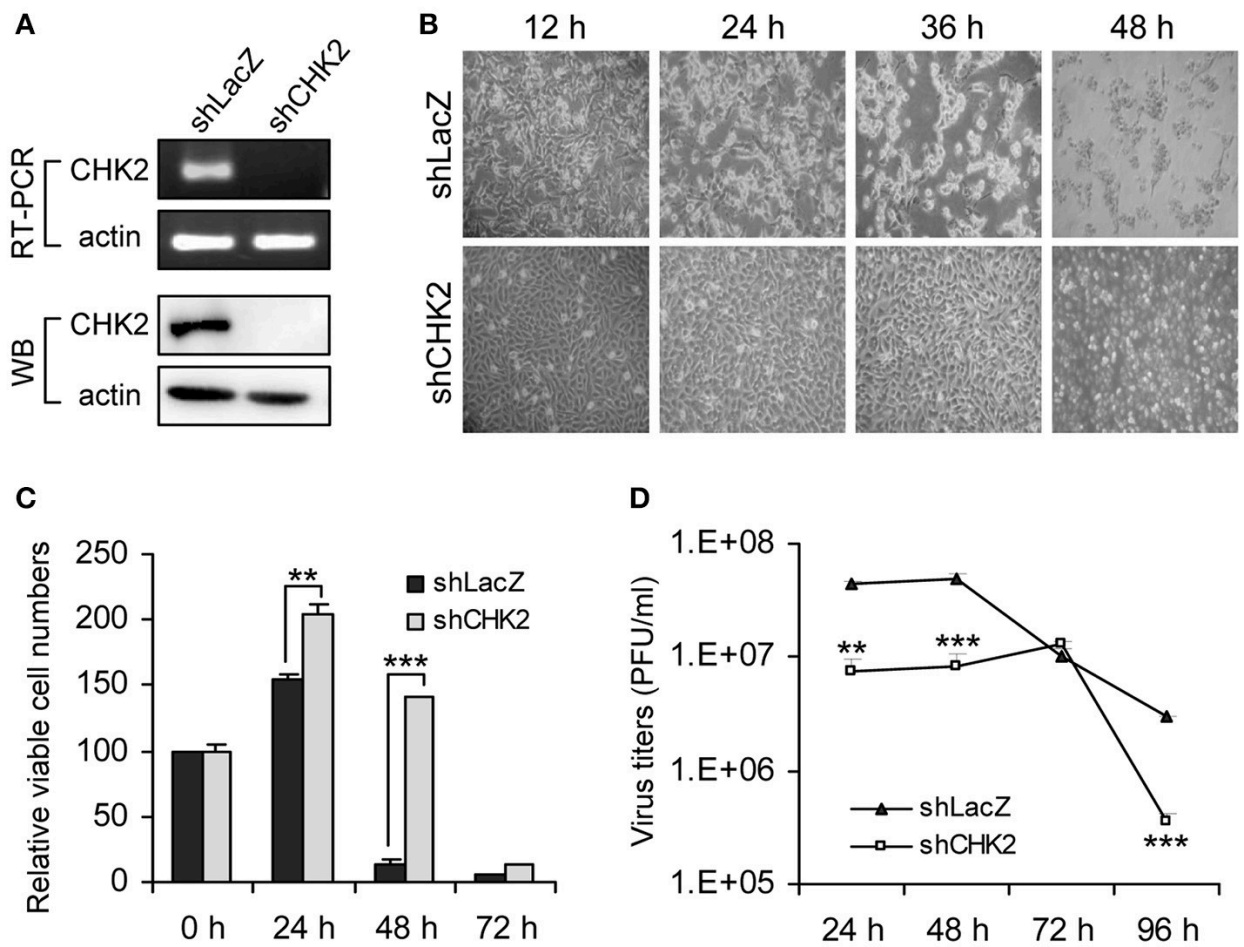
Human kinase/phosphatase-wide RNAi screening identified CHK2 as a cellular factor involved in JEV infection. **(A)** Human U87 cells transduced with lentivirus expressing shRNA targeting CHK2 (shCHK2) or LacZ control (shLacZ) were verified for CHK2 knockdown by RT-PCR for mRNA level and immunoblotting analysis for protein expression. The cells were infected with JEV (MOI 5) for the indicated times. Cell morphology **(B)**, relative viable cell numbers (*n* = 3) were determined by trypan blue exclusion **(C)** and viral progeny production (*n* = 3) was determined by plaque-forming assays **(D)**. Data are mean ± SD. ^**^p < 0.01; ^***^p < 0.005 by two-tailed Student *t-*tests.

**Figure 3:**
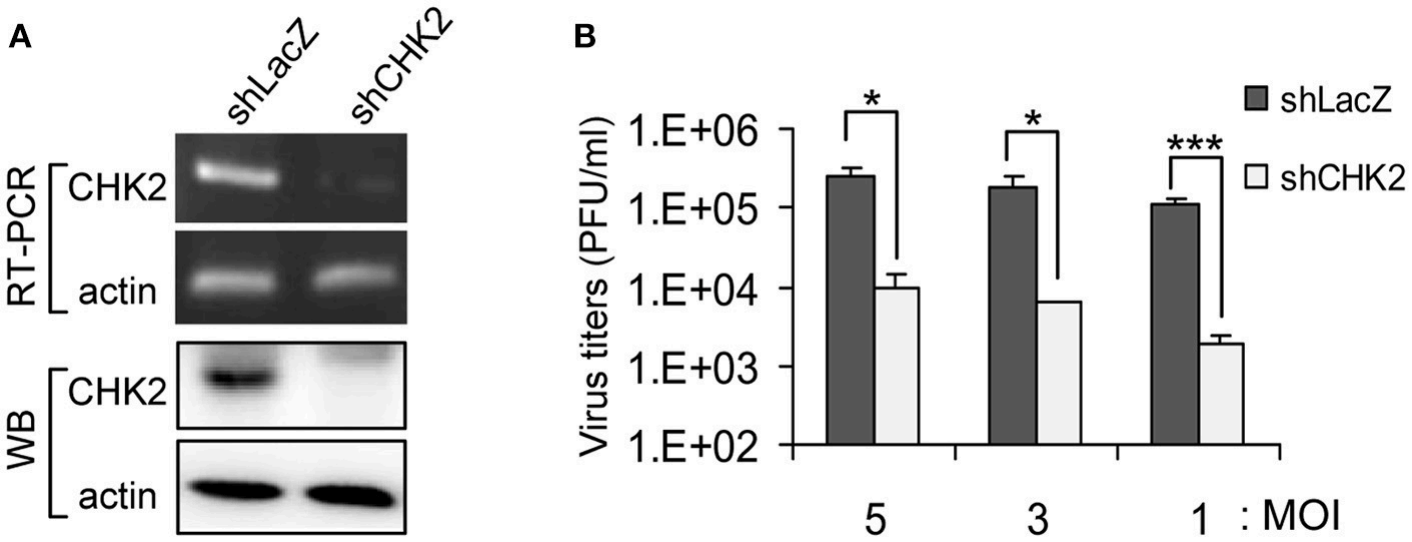
Reduced CHK2 expression decreases JEV replication. Human A549 cells were transduced with shCHK2-expressing lentivirus and selected by puromycin. ShRNA-targeting LacZ was the non-targeting sequence control. **(A)** The RNA and protein levels of CHK2 were verified by RT-PCR with CHK2-specific primers and immunoblotting with anti-CHK2 antibody, respectively. Cells were infected with JEV at the indicated MOIs (5, 3, and 1); **(B)** at 24 h post infection, culture supernatants were collected for virus titration by plaque-forming assays. Data are mean ± SD virus titers (*n* = 3). ^*^p < 0.05; ^***^p < 0.005 by two-tailed Student *t-*test.

To verify whether knockdown of these candidate genes indeed rescued cells from JEV infection, we transduced U87 cells with the lentiviral vector targeting each candidate gene and infected the cells with JEV. Knockdown of one of these candidate genes, CHEK2, substantially rendered cell survival from JEV infection. U87 cells showed reduced expression of CHK2 by transduction with lentivirus expressing an shRNA targeting CHK2 (Figure 2A). Upon JEV infection, knockdown of CHK2 resulted in reduced CPE (Figure 2B), enhanced cell survival (Figure 2C) and reduced JEV progeny production (Figure 2D) as compared with control knockdown shLacZ cells. To ascertain the importance of CHK2 in JEV infection, we further checked the involvement of CHK2 in another human cell line, A549 cells. Similarly, JEV production was reduced in human A549 cells with knocked-down CHK2 expression (Figure 3).

### JEV-activated CHK2 is beneficial for JEV replication

**Figure 4:**
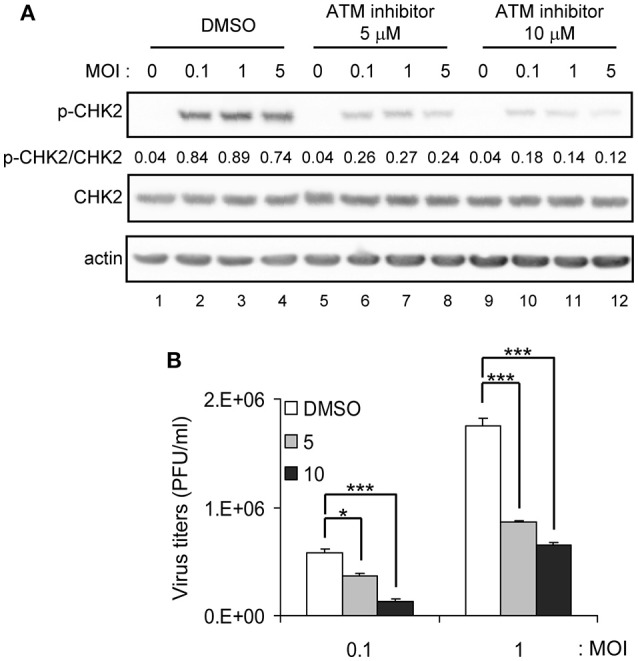
ATM inhibitor reduces JEV replication. A549 cells were infected with JEV at MOI 0.1, 1 or 5 in the absence (DMSO solvent control) or presence of 5 or 10 μM ATM inhibitor (KU-55933) as indicated. **(A)** Immunoblotting analysis of cell lysates at 24 h with antibodies against T68-phosphorylated CHK2, CHK2 and actin. **(B)** A549 cells were treated with ATM inhibitor (KU-55933) at the indicated dose, followed by JEV infection at MOI 0.1 or 1 for 24 h. Culture supernatants were collected for virus titration by plaque-forming assays. Data are mean ± SD virus titers (*n* = 3). ^*^p < 0.05; ^***^p < 0.005 by two-tailed Student *t-*test.

CHK2 is a protein kinase activated by ATM in response to DNA damage (Bakkenist and Kastan, 2004). To understand how CHK2 participates in JEV replication, we determined whether the ATM/CHK2 pathway was activated by JEV infection. CHK2 activation, as manifested by phosphorylation at Thr-68, was evident in JEV-infected cells in an MOI dependent manner, and an ATM inhibitor KU-55933 (Lau et al., 2005) reduced the CHK2 activation (Figure 4A). Furthermore, the ATM inhibitor significantly reduced JEV production (Figure 4B).

**Figure 5:**
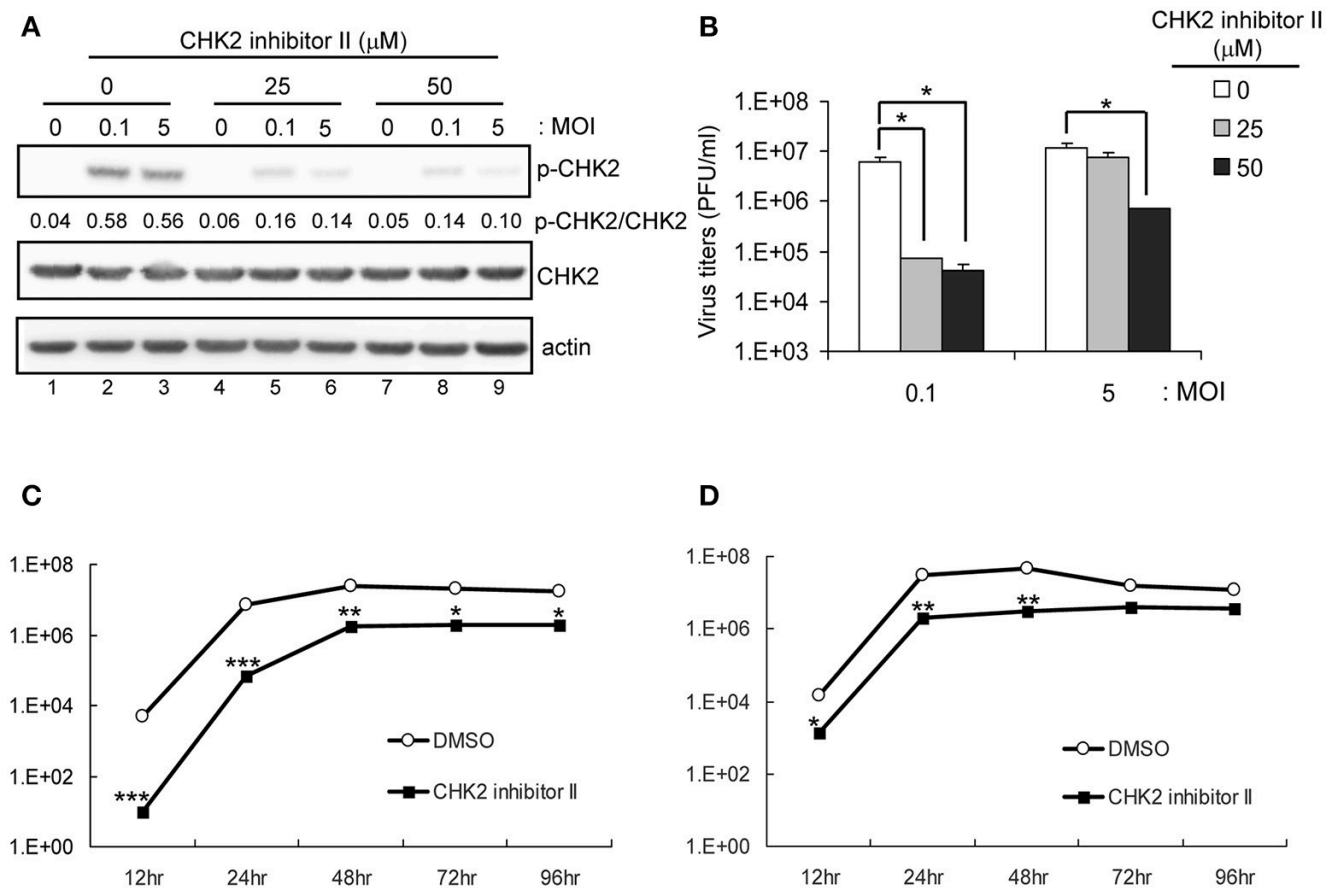
Inhibition of CHK2 activity reduces JEV replication in human U87 glioma cells. U87 cells were infected with JEV (MOI 0, 0.1 and 5) with or without of CHK2 inhibitor II (0, 25 and 50 μM). **(A)** At 24 h post treatment, immunoblotting analysis of cell lysates at 24 h with antibodies against, T68-phosphorylated CHK2, CHK2, and actin was performed; virus titration **(B)** in culture supernatant was determined by plaque-forming assays. Cells were treated with DMSO or CHK2 inhibitor II (50 μM), followed by JEV infection at MOIs of 0.1 **(C)** or 5 **(D)** for the indicated times. Culture supernatants were collected for virus titration by plaque-forming assays. Data are mean ± SD virus titers (*n* = 3). ^*^p < 0.05; ^**^p < 0.01; ^***^p < 0.005 by two-tailed Student *t-*test.

**Figure 6:**
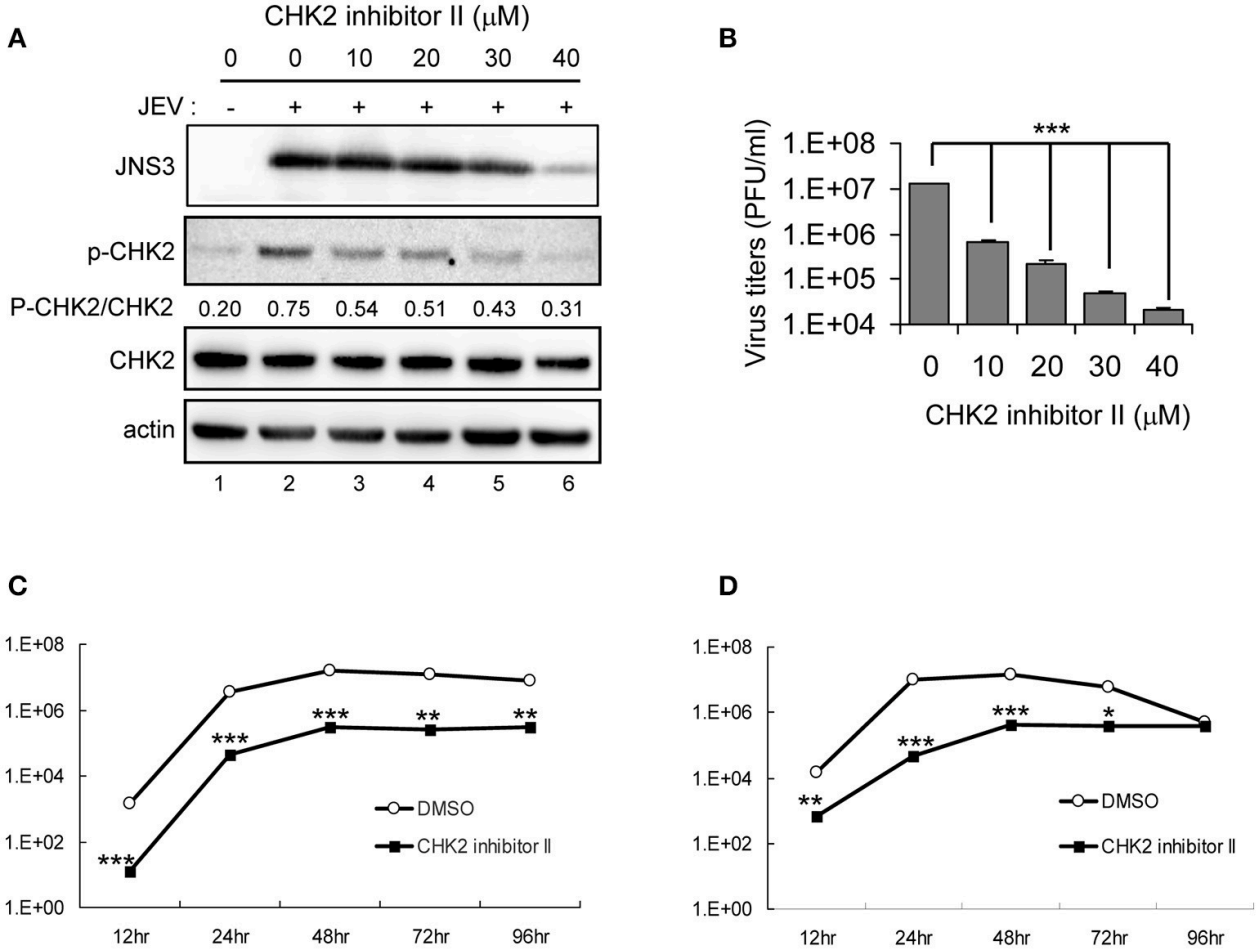
Inhibition of CHK2 activity reduces JEV replication in human A549 lung carcinoma cells. A549 cells were mock-infected or infected with JEV (MOI 5) with various doses of CHK2 inhibitor II. **(A)** At 24 h post treatment, immunoblotting analysis of cell lysates at 24 h with antibodies against JEV NS3, T68-phosphorylated CHK2, CHK2, and actin and was performed; **(B)** virus titers determination in culture supernatants were determined by plaque-forming assays. Cells were treated with DMSO or CHK2 inhibitor II (50 μM), followed by JEV infection at MOIs of 0.1 **(C)** or 5 **(D)** for the indicated times. Culture supernatants were collected for virus titration by plaque-forming assays. Data are mean ± SD virus titers (*n* = 3). ^*^p < 0.05; ^**^p < 0.01; ^***^p < 0.005 by two-tailed Student *t-*test.

**Figure 7:**
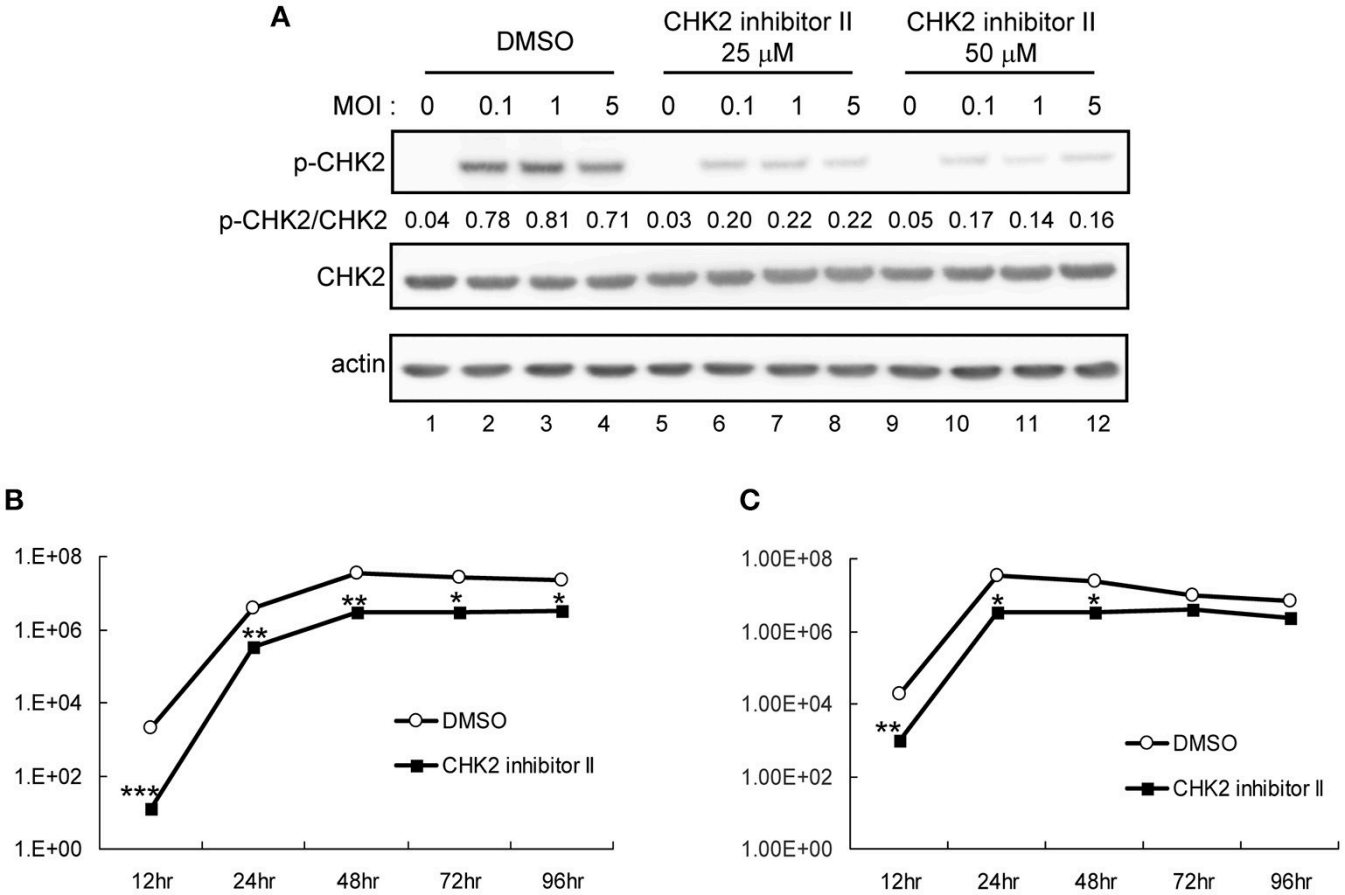
Inhibition of CHK2 activity reduces JEV replication in human BE(2)C neuroblastoma cells. BE(2)C cells were infected with JEV at MOI 0.1, 1 or 5 in the absence (DMSO solvent control) or presence of 25 or 50 μM CHK2 inhibitor II as indicated. **(A)** Immunoblotting analysis of cell lysates at 24 h with antibodies against T68-phosphorylated CHK2, CHK2 and actin. BE(2)C cells were treated with DMSO or CHK2 inhibitor II (50 μM), followed by JEV infection at MOIs of 0.1 **(B)** or 5 **(C)** for the indicated times. Culture supernatants were collected for virus titration by plaque-forming assays. Data are mean ± SD virus titers (*n* = 3). ^*^p < 0.05; ^**^p < 0.01 ^***^p < 0.005 by two-tailed Student *t-*test.

The involvement of CHK2 in JEV replication was further assessed by using a cell-permeable CHK2 inhibitor, CHK2 inhibitor II (Arienti et al., 2005). U87 cells were infected with JEV (MOI 0.1 and 5) in the absence or presence of non-cytotoxic CHK2 inhibitor II (25 and 50 μM). Immunoblotting analysis with anti-CHK2 (Thr68) antibody showed that CHK2 inhibitor II effectively reduced the JEV induced phosphorylation of CHK2 protein (Figure 5A). CHK2 inhibitor II also reduced virus production. With low MOI, 25 μM CHK2 inhibitor II readily decreased JEV production by ~100-fold (Figure 5B). With high MOI, 25 μM CHK2 inhibitor II slightly suppressed JEV production, whereas 50 μM conferred 94% reduction in virus production. With the treatment of 50 μM CHK2 inhibitor II, virus progeny was inhibited in both high MOI (Figure 5C) and low MOI (Figure 5D) at various time points. The antiviral effect of the CHK2 inhibitor II was further verified in A549 cells and BE(2)C cells. CHK 2 inhibitor II reduced both the viral protein expression and CHK2 phosphorylation level (Figure 6A) Viral progeny production (Figure 6B) was suppressed by CHK2 inhibitor II at different time points (Figures 6C, D). Similar to U87, the CHK2 phosphorylation of BE(2)C neuroblastoma cells was induced by JEV and reduced by CHK2 inhibitor II (Figure 7A). The virus progeny in both high MOI (Figure 7B) and low MOI (Figure 7C) was inhibited by 50 μM CHK2 inhibitor II in various time points. Collectively, blocking the ATM/CHK2 pathway by an ATM inhibitor or a CHK2 inhibitor reduced JEV replication in human glioblastoma, lung cancer carcinoma, and neuroblastoma cell lines tested.

### JEV infection resulted in G1 arrest in U87, A549, BE(2)C cells

**Figure 8:**
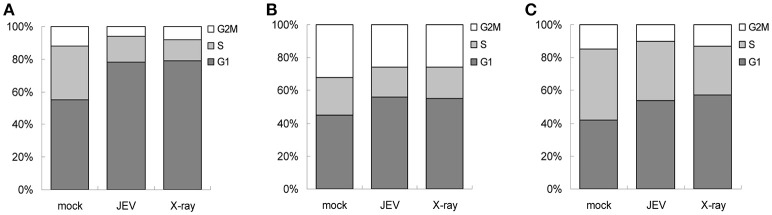
JEV infection induces cell cycle arrest at G1 phase. U87 **(A)**, A549 **(B)**, and BE(2)C **(C)** cells were mock-infected, JEV (MOI 5) infected, or X-ray (8 Gy) treated. At 24 h post-treatment, DNA content was determined by PI staining and analyzed by flow cytometry for proportion of cells in G1, S, and G2 phases.

Given that the ATM/CHK2 pathway is activated upon DNA damage in order to modulate cell cycle progression, we assessed whether JEV infection results in cell cycle arrest. We analyzed the cell cycle by measuring DNA content with PI staining for mock- and JEV-infected cells at 24 h post-infection. JEV infection increased the proportion of cells in the G1 phase from 55.46% to 78.87% in U87 cells (Figure 8A). Similar G1 phase arrest was also noted in A549 cells (Figure 8B) and BE(2)C cells (Figure 8C). Therefore, JEV infection induced cell cycle arrest in the G1 phase.

## Discussion

With the recent development of screening strategies, RNAi-based technology has been extensively used to identify genes involved in cellular processes, such as signal transduction, cell cycle, cancer biology and host–pathogen interactions (Houzet and Jeang, 2011). Viruses are obligate intracellular parasites that may utilize host cell machineries during every step of their life cycles (Goff, 2007). In this study, we used a human kinase/phosphatase-wide RNAi strategy for large scale screening to identify a gene(s) involved in regulating JEV infection in U87 human glioma cells. Among the 1260 human kinases/phosphatases found, we identified a cell cycle-regulating molecule, CHK2, that facilitated JEV infection in the cells.

For infectivity, viruses have been found to interact with CHK2 and its related responses in host cells. Human papillomaviruses activate the ATM DNA response (Lai et al., 2008; Moody and Laimins, 2009). HSV-1 and SV40 recruit members of the ATM DNA damage pathway to specific sites in the nucleus during replication (Taylor and Knipe, 2004; Wilkinson and Weller, 2004; Zhao et al., 2008). Furthermore, the NS5B of HCV was found to interact with both ATM and CHK2, and the replication of subgenomic replicon RNA of HCV was suppressed in cells with CHK2 knockdown (Ariumi et al., 2008). In this study, we also found that downregulation of CHK2 and inhibition of CHK activity enhanced cell resistance to JEV infection. Likewise, progeny virus production and expression of viral proteins were reduced in cells with knockdown of functional CHK2. Because viral replication induces cellular stresses and triggers an ATR response, viruses may hijack CHK2 to elongate the cell cycle arrest, thus creating a time window for viral replication. Consequently, downregulation of CHK2 becomes a suitable way to inhibit virus propagation.

Viruses are streamlined organisms featured in minimizing proteins required for genome replication and control of the host cell cycle. In analogy to some RNA viruses such as influenza A virus (He et al., 2010), mouse hepatitis virus (Chen and Makino, 2004), and severe acute respiratory syndrome coronavirus (Yuan et al., 2007), JEV infection is able to regulate cell-cycle arrest in the G1 phase, probably resulting from CHK2 activation (Lavin and Kozlov, 2007). Upon activation, CHK2 phosphorylates Cdc25a and Cdc25c (phosphatases), which results in G1/S or G2/M arrest via degradation or cytoplasmic sequestration, respectively (Antoni et al., 2007). Each phase of the cell cycle should represent a unique metabolic status. Indeed, the transcription activity of Pol II is much higher in the G1 phase than in the S or G2/M phase (Yonaha et al., 1995). As well, in non-dividing hepatocytes, the G0/G1 state has higher translation efficiency, which facilitates expression of the HCV genome (Fehr et al., 2012). Likewise, to proliferate the viral genome, JEV may arrest the cell cycle in the G1 phase to increase Pol II transcription activity and translation efficiency. Moreover, oncogenic viruses often possess mechanisms to induce G1/S cell cycle arrest via p53 inactivation, thus rescuing the infected cells from apoptosis (Yew and Berk, 1992; Scheffner et al., 1993; Härtl et al., 2008). JEV inducing G1 arrest may be advantageous to gain sufficient time and resources for viral replication and to avoid early apoptosis of infected cells.

Although the exact mechanism of how JEV induces the DNA damage response is not fully understood, our studies demonstrate that JEV executes its own replication by manipulating the host cell cycle via CHK2. The kinase/phosphatase-wide RNAi screening system can be an effective strategy to search for cellular factors involved in the regulation of JEV infection.

## Author contributions

Y-LC and Y-LL designed research, analyzed data and wrote the paper. Y-LC performed research. C-LL provided critical reagents.

### Conflict of interest statement

The authors declare that the research was conducted in the absence of any commercial or financial relationships that could be construed as a potential conflict of interest.
